# Micronucleus-specific histone H1 is required for micronuclear chromosome integrity in *Tetrahymena thermophila*

**DOI:** 10.1371/journal.pone.0187475

**Published:** 2017-11-02

**Authors:** Juxia Qiao, Jing Xu, Tao Bo, Wei Wang

**Affiliations:** 1 Key Laboratory of Chemical Biology and Molecular Engineering of Ministry of Education, Institute of Biotechnology, Shanxi University, Taiyuan, Shanxi, China; 2 College of Life Science, Shanxi University, Taiyuan, Shanxi, China; 3 Institute of Evolution & Marine Biodiversity, Ocean University of China, Qingdao, Shandong, China; Washington University in Saint Louis, UNITED STATES

## Abstract

Histone H1 molecules play a key role in establishing and maintaining higher order chromatin structures. They can bind to linker DNA entering and exiting the nucleosome and regulate transcriptional activity. *Tetrahymena thermophila* has two histone H1, namely, macronuclear histone H1 and micronuclear histone H1 (Mlh1). Mlh1 is specifically localized at micronuclei during growth and starvation stages. Moreover, Mlh1 is localized around micronuclei and forms a specific structure during the conjugation stage. It co-localizes partially with spindle apparatus during micronuclear meiosis. Analysis of *MLH1* knock-out revealed that Mlh1 was required for the micronuclear integrity and development during conjugation stage. Overexpression of Mlh1 led to abnormal conjugation progression. RT-PCR analysis indicated that the expression level of *HMGB3* increased in Δ*MLH1* strains, while the expression level of *MLH1* increased in Δ*HMGB3* cells during conjugation. These results indicate that micronuclear integrity and sexual development require normal expression level of Mlh1 and that HmgB3 and Mlh1 may functionally compensate each other in regulating micronuclear structure in *T*. *thermophila*.

## Introduction

Chromatin in eukaryotic cells consists of DNA, histones, non-histones and RNA. The nucleosome is the basic unit of chromatin with 146 bp of DNA wrapped around core histone octamer consisting of two copies of evolutionarily conserved core histones, namely H2A, H2B, H3, and H4 [1]. Histone H1 and non-histone chromosomal proteins bind to DNA between nucleosomes [2]. Histone H1 is variable across organisms and mediates gene-specific regulation of transcription as well as DNA-dependent processes [3–5]. H1 also provides a platform for chromatin remodeling and efficient repair of DNA damage [6]. Multiple histone H1 family members are present in different organisms. Eleven H1 variants have been found in mammals, five in *Xenopus laevis* and eight in *Caenorhabditis elegans*, but only one linker histone H1 has been identified in *Physarum polycephalum* and *Drosophila melanogaster* [5, 7, 8]. In higher eukaryotes, most H1 variants have similar structure, comprising primarily of an N-terminal region, a C-terminal tail, and a central conserved globular region [9]. In *Saccharomyces cerevisiae*, Hho1p contains two regions of sequence homology to the central globular domain of the canonical histone H1 [10].

*Tetrahymena* has two different nuclei, a macronucleus (Mac) and a micronucleus (Mic). Mac divides amitotically and is transcriptionally active, while Mic divides mitotically and is transcriptionally inactive at vegetative stages. The macronuclear histone H1 is smaller, more basic, and lacks a conserved globular domain [11, 12]. Functionally, macronuclear histone H1 is not essential for chromatin packaging, condensation and cellular viability [13]. However, it can regulate the expression of specific genes [14]. Micronucleus-specific histone H1 (Mlh1) is different from macronuclear H1 and H1 from other organisms [15]. Mlh1 is larger and is proteolytically processed into different components (α, β, γ, and δ) [16]. Both β and γ resemble Hl histones but lack a globular domain [13]. δ contains two high mobility group (HMG) boxes, while α is simply the uncleaved fusion of δ and γ [16].

Knocking out *MLH1* does not affect *Tetrahymena* viability and cellular survival during vegetative growth stage, but causes enlarged Mic. Macronuclear histone H1 knock-out cells have enlarged Mac and normal-sized Mic [13]. However, the function of Mlh1 during sexual development is unclear. In the present study, we focused on the localization and function of Mlh1 during sexual development. We found that Mlh1 maintained micronuclear chromosome integrity and thus played an important role in *Tetrahymena* conjugation.

## Materials and methods

### *Tetrahymena* strains and culture conditions

The B2086 and CU428 *T*. *thermophila* strains were provided by P. J. Bruns (Cornell University, now available at the National *Tetrahymena* Stock Center, http://tetrahymena.vet.cornell.edu/index.html). Cells were cultured in super proteose peptone medium (1% proteose peptone, 0.1% yeast extract, 0.2% glucose, and 0.003% EDTA ferric sodium salt) at 30°C for vegetative growth [17]. Two different mating type cells were washed, starved (18 to 24 h, without shaking at 30°C), and mixed in 10 mM Tris-HCl (pH 7.4) at equal quantities (2.0–3.0×10^5^ cells/ml) for conjugation [18].

### Creation of HA-*MLH1* strains

To create HA-tagged *MLH1* constructs, *MLH1* and *MLH1* fragments (α, β, γ, and δ) were amplified by PCR using the genomic DNA isolated from B2086 cells using specific primers (S1 Table). These PCR products were cloned into pMD18-T vector and confirmed by sequencing. Then they were digested with *BamH* I and *Asc* I, and ligated with pXS75 vector digested with the same enzyme. The recombinant plasmids were subsequently digested with *Sac* I/*Xho* I. The fragments were introduced into the CU428 and B2086 strains using biolistic particle transformation system GJ-1000 (SCIENTZ, China). Tansformants were selected on the basis of resistance to paromomycin and identified by PCR using the *MTT1*-FW/*MTT1*-RV primer set, as described previously [19].

### Generation of somatic *MLH1* knock-out strains

*MLH1* (TTHERM_00471820) macronuclear genomic and mRNA sequences were obtained from the *Tetrahymena* Genome Database (http://www.ciliate.org) and *Tetrahymena* Functional Genomics Database (TetraFGD, http://tfgd.ihb.ac.cn) [20, 21]. To generate a *MLH1* knock-out construct, 3′ and 5′ flanking sequences of *MLH1* were amplified from *Tetrahymena* genomic DNA using primers KO-*MLH1*-3′-FW/KO-*MLH1-*3′-RV and KO-*MLH1-*5′-FW/KO-*MLH1-*5′-RV (S1 Table), respectively. 3′ and 5′ flanking sequence of *MLH1* were inserted into downstream and upstream sequence of Bsr cassette (consisting of the *HHF1* promoter, the blasticidin S resistance gene *BSR*, and *BTU2* 3′ flanking sequence and conferring blasticidin resistance) in pBsr, respectively. B2086 and CU428 cells were transformed with the construct pBsr-*MLH1* using a biolistic particle transformation system GJ-1000 (SCIENTZ, China) [22]. Somatic *MLH1* knock-out cells were selected in increasing concentrations of blasticidin, starting from 100 μg/ml to a final concentration of 1600 μg/ml until the endogenous macronuclear *MLH1* gene was completely replaced by phenotypic assortment.

### Micronuclear integrity assay

Micronuclear chromosomes contain micronuleus-specific chromosome breakage sequences (Cbs). Using 5 pairs of specific primers flanking Cbs, which were from different chromosomes 1L-4, 2R-1, 3L-2, 4L-2, 5–1, Mic chromosome integrity was analyzed by PCR [23]. The PCR temperature cycling conditions were: 5 min at 94°C, 45 cycles of 30 s at 94°C, 30 s at 50°C (primers of II) or 30 s at 54°C (primers of I, III, IV, and V), 2 min at 68°C, and 10 min at 68°C.

### Indirect immunofluorescence

Mating cells (5 ml, 2.5×10^5^ cells/ml in 10 mM Tris, pH7.4) were fixed with 20 μl of Schaudinn’s fixative (2:1, saturated HgCl_2_:100% ethanol,) for HA or α-tubulin antibody or 5 ml of Lavdowsky’s fixative (50:39:10:1, ethanol:water:formalin:acetic acid) for γ-H2A.X antibody overnight. Fixed cells (50 μl) were immobilized on cover glasses coated with poly-L-lysine (Sigma) and dried for 30 min at 30°C. The fixed cells were washed with PBS and PBST (0.05% Triton X-100 or 0.1% Tween-20) three times, each 10 min, Then, the cells were incubated with a blocking buffer (3% bovine serum albumin,10% goat serum, and 0.4% Triton X-100) for 1 h at 37°C, followed by incubation with anti-γ-H2A.X antibody (1:200, Beyotime) for 2 h at RT or anti-HA antibody (1:1000, Millipore) and anti-α-tubulin antibody (1:2000, Millipore) overnight at 4°C. The cells were then washed and incubated with fluorescein isothiocyanate (FITC)-conjugated donkey anti-mouse (1:800, Abcam) or FITC-conjugated goat anti-rabbit IgG antibody (1:2000, Millipore) for 1 h at RT. DNA were stained with 1 μg/ml 4’, 6-diamidino-2-phenylindole dihydrochloride (DAPI) for 10 min at RT. Digital images were acquired with the Delta Vision Elite deconvolution microscope system (Applied Precision/GE Healthcare) or a fluorescence microscopy (FV1000, OLYMPUS, Japan). Brightness and contrast of images were adjusted using Adobe Photoshop software [24, 25].

### Isolation of total cellular RNA

Trizol and organic solvent were used to extract the total RNA of cells, which was followed by reverse transcription using PrimeScript^TM^ RT reagent Kit with gDNA Eraser (Perfect Real Time) (TaKaRa). RNA samples were pretreated to eliminate genomic DNA with gDNA Eraser at 42°C for 2 min, then reverse-transcription was conducted with PrimeScript RT Enzyme Mix 1 for 15 min at 37°C, and 85°C for 5 s, and maintained at 4°C.

### RT-PCR

RT-PCR was performed with the SYBR Premix Ex TaqTM (TaKaRa) on an ABI StepOne Plus system (Applied Biosystems, USA) [26]. The following parameters were progressively used for PCR before the dissociation stage and with 17S rRNA as the internal control: 30 s at 95°C, 40 cycles of 95°C for 5 s, 60°C for 35 s. Data from the real-time PCR experiments were analyzed using the 2^−ΔΔCt^ method. Each reaction was performed in triplicate using RT-*HMGB*-FW/RT-*HMGB3*-RV and RT-*MLH1*-FW/RT-*MLH1*-RV as the primers (S1 Table).

### Immunoblotting

Histones were isolated from log phase of WT strains and mutants using total histone extraction kit (Epigentek, USA), then separated by 12% SDS-PAGE (sodium dodecyl sulfate-polyacrylamide gel electrophoresis) and transferred to PVDF membranes (110 V, 3.5 h). The membranes were blocked in 5% nonfat milk and incubated with anti-HA (1:300, Millipore) primary antibody overnight at 4°C. Following three washes, the membranes were incubated with HRP-conjugated goat anti-rabbit IgG antibody (1:800, Millipore) for 1 h at 37°C. Finally, blots were developed with Western Blotting Detection Reagent (Engreen, China) according to the product instructions.

## Results

### Identification and analysis of *MLH1* in *Tetrahymena*

**Fig 1:**
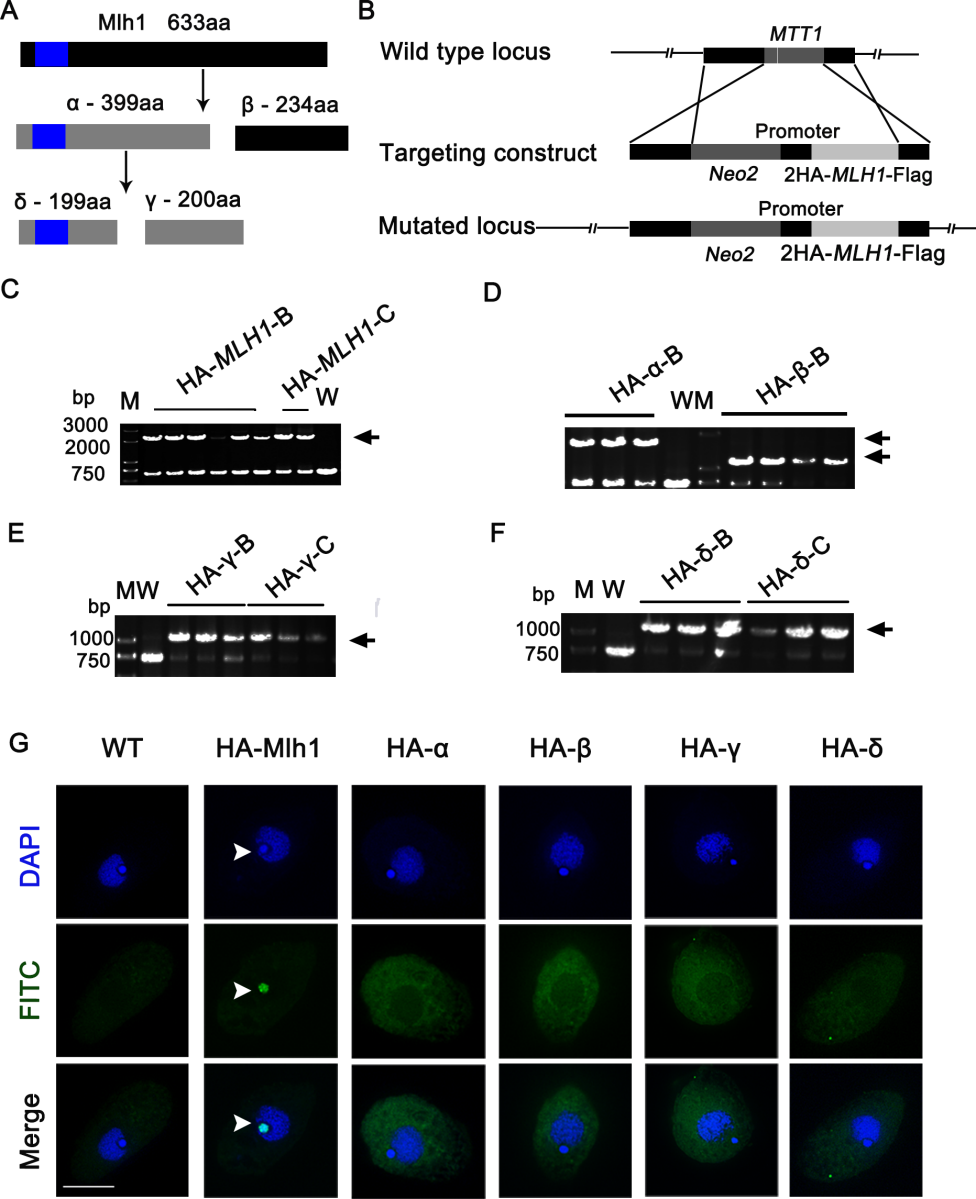
Mlh1 specifically localized at Mic. (A) The diagram of Mlh1 and its hydrolyzed products, blue rectangle indicates HMG-box [16]. (B) Schematic drawings of the *MTT1* locus and the HA-*MLH1* knock-in construct in which the *MTT1* coding sequence was replaced by HA-*MLH1*. (C) Identification of HA-*MLH1* mutants where the total DNA was isolated from wild type CU428, HA-*MLH1*-B, and HA-*MLH1*-C cells. The recombinants were identified by PCR. The arrow indicates recombination band (~2.2 kb). (D) Identification of HA-α-B and HA-β-B mutants. Total DNA was isolated from wild-type CU428, HA-α-B, and HA-β-B cells. The recombinants were identified by PCR. Arrow indicates the recombination band (HA-α~1.5 kb; HA-β~1.0 kb). (E) Identification of HA-γ mutants. Total DNA was isolated from wild-type CU428, HA-γ-B and HA-γ-C cells. The recombinants were identified by PCR. The arrow indicates recombination band (~0.9 kb). (F) Identification of HA-δ mutants. Total DNA was isolated from wild-type CU428, HA-δ-B, and HA-δ-C cells. The recombinants were identified by PCR. The arrow indicates recombination band (~0.9 kb). (G) Localization of HA-Mlh1, HA-α, HA-β, HA-γ and HA-δ during the growth stages. HA-tagged proteins were identified by anti-HA antibody and DNA was stained with DAPI. Scale bar, 10 μm.

Based on the *Tetrahymena* Genome Database (http://www.ciliate.org), bioinformatic analysis showed that *MLH1* is a single copy and has no intron, with an open reading frame of 1902 bp encoding 633 amino acids. Mlh1 contains 21.5% lysine and 23.1% serine. Primary sequence analysis of Mlh1 shows that it has no similarities with other H1 histones from other organisms. It contains two conserved HMG-box domain (93–158 amino acids), which belongs to class II/III members of the HMG-box superfamily. *Tetrahymena* Functional Genomics Database (TetraFGD, http://tfgd.ihb.ac.cn) shows that *MLH1* has a similar expression profile as *HMGB3* [21, 27]. Furthermore, HmgB3 and Mlh1 contain similar numbers of charged and hydrophobic amino acids, and share the conserved HMG box domain [19]. Thus, Mlh1 and HmgB3p could have similar functions in *Tetrahymena*. Linker histone phosphorylation has been suggested to participate in both chromosome condensation and transcriptional regulation. In *Tetrahymena*, macronuclear Histone H1 is highly phosphorylated at serine or threonine residues located in a cdc2 kinase consensus motif [28]. HmgB3 is also phosphorylated at 7 different residues [19]. Although phosphoproteomics analysis of *Tetrahymena* showed that Mlh1 has no phosphorylated modification,Wu M. *et al* showed that Mlh1 contains 16 protein kinase A phosphorylation sites [16]. Furthermore, Mlh1 was processed into four different components α, β, γ, and δ (Fig 1A), each product has at least one canonical phosphorylation site for protein kinase A [16].

### Localization of HA-Mlh1 during different developmental stages

The localization pattern of the H1s is closely involved in their function [29]. To analyze localization of Mlh1, we first created HA-*MLH1* strains, with HA coding sequence inserted at the N terminus of the *MLH1* open reading frame. HA-Mlh1 was expressed under the control of *MTT1* promoter with Cd^2+^ induction (Fig 1B). HA-*MLH1* partially replaced the *MTT1* gene (Fig 1C). Similarly, HA-α, HA-β, HA-γ, and HA-δ also partially replaced the *MTT1* gene in these mutants, respectively (Fig 1D–1F). Immunostaining showed that HA-Mlh1 specifically localized at the Mic in the growing cells. However, HA-α, HA-β, HA-γ, and HA-δ localized in the cytoplasm (Fig 1G). Western blot analysis showed that HA-Mlh1, HA-α, HA-β, HA-γ, and HA-δ were expressed in the mutant cells, respectively (S1 Fig). HA-α, HA-β, HA-γ, and HA-δ were expressed at levels similar to HA-Mlh1. The results indicated their absence in Mic is not an expression artifact, but due to mis-localization. Although full length Mlh1 is partially degraded raises the possibility that it is a degradation product that localizes to the Mic, we also found that similar sized degradation products are detected in expression of HA-α mutant cells (S1 Fig). So, it appears that α, β, γ, and δ from cytoplasm failed to transport into Mic during the vegetative growing stage. The full length Mlh1 was transported into the Mic and hydrolyzed into different fragments in Mic.

**Fig 2:**
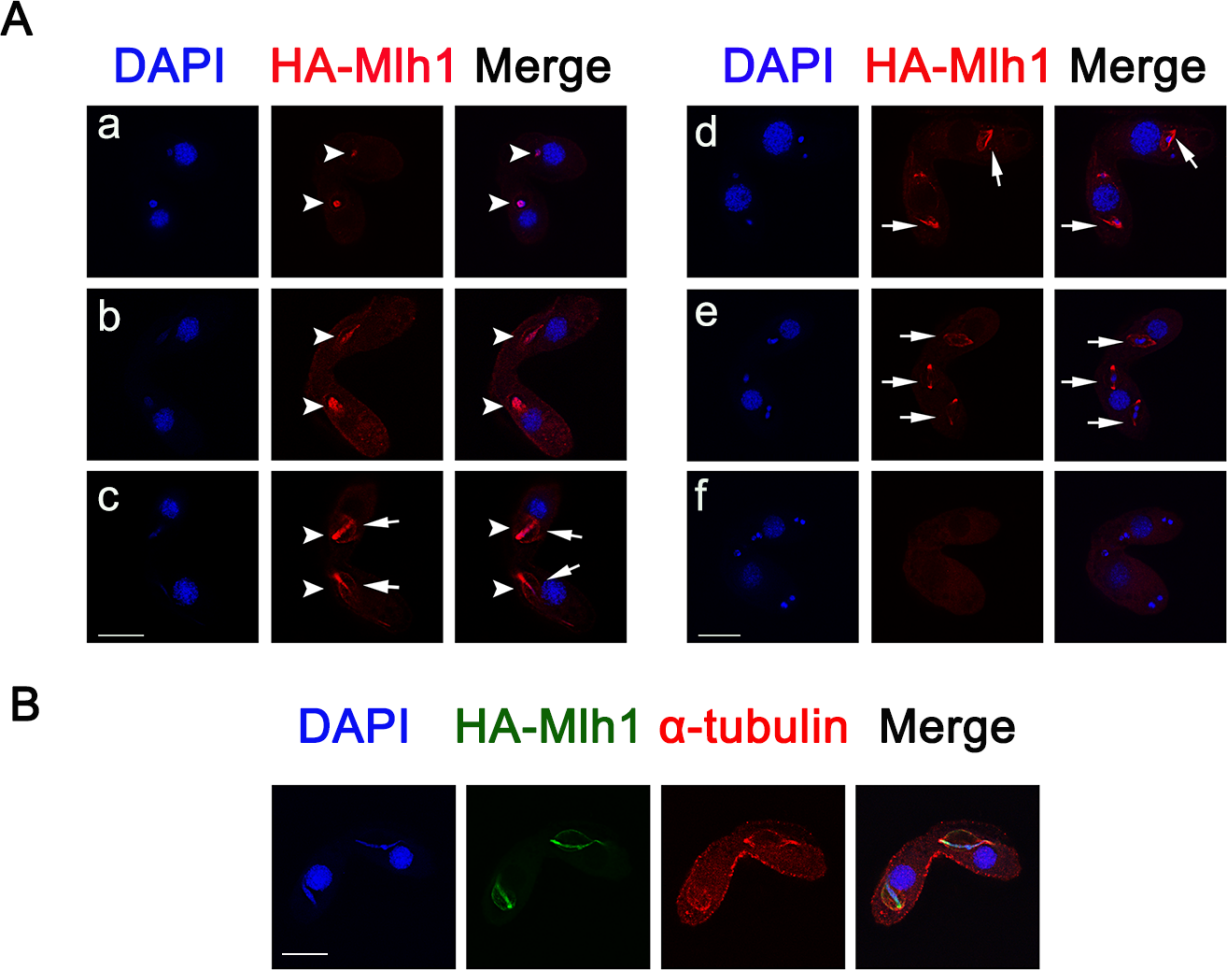
Localization of Mlh1 during conjugation stage. (A) Localization of HA-Mlh1 during the conjugation stage. The cell was induced by Cd^2+^ in the growing stage. HA-Mlh1 was detected by anti-HA antibody and DNA was stained with DAPI. a, pair formation; b, early crescent; c, crescent; d, meiosis I; e, meiosis II; f, pronuclear selection. Arrowheads indicate Mics. Arrows show spindle-shaped micronuclei. Scale bar, 10 μm. (B) Mlh1 co-localized with spindle structures during the crescent stage. HA-Mlh1 was detected using anti-HA antibody, spindle structure was detected with anti α-tubulin antibody, and DNA was stained with DAPI. Scale bar, 10 μm.

During the early mating stages, HA-Mlh1 localized at the Mic (Fig 2Aa–2Ac). The HA-Mlh1 signal covered more than the DAPI-stained areas in Mic. Interestingly, the localization signal of Mlh1 is similar to that of the spindle apparatus (Fig 2Ad and 2Ae), implying that Mlh1 may be involved in spindle function. Consistent with this idea, we found Mlh1 co-localized with α-tubulin signal around separating micronuclei during the crescent stage (Fig 2B). HA-Mlh1 signal disappeared after the completion of Mic meiosis (Fig 2Af).

### Knocking out *MLH1* affects Micronuclear DNA stability

**Fig 3:**
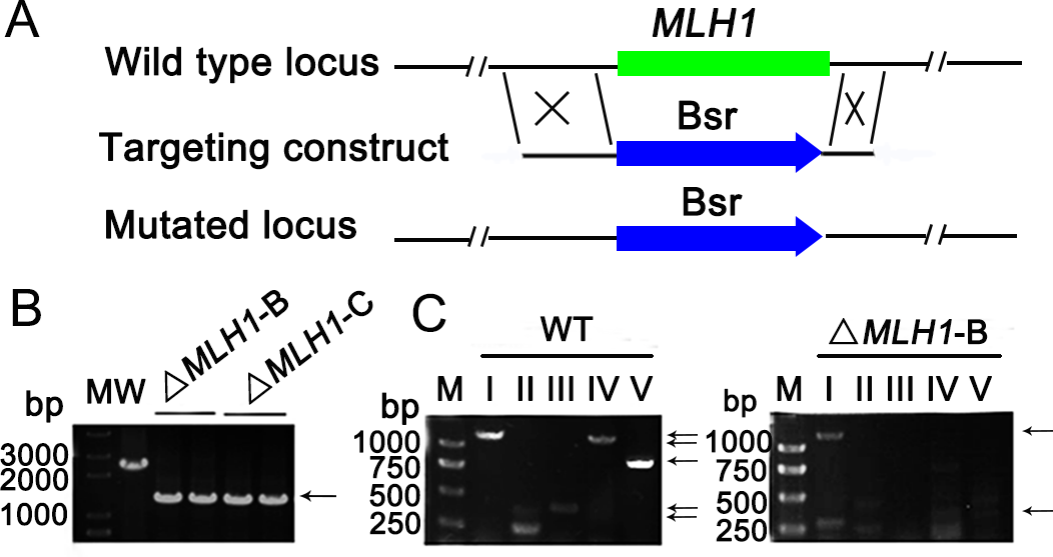
Knocking out *MLH1* affects DNA stability. (A) Schematic drawings of the *MLH1* locus and the knock-out construct used to disrupt it. *MLH1* was replaced by Bsr cassette by homologous recombination. (B) Identification of Δ*MLH1*. Total DNA was isolated from wild-type CU428 and somatic *MLH1* knock-out cells. Δ*MLH1-*B and Δ*MLH1-*C are different mating type mutants from B2086 and CU428 respectively. Different lanes represent different clones. The target gene was amplified by PCR. The arrow indicates recombination band (~1.4 kb). (C) Mic-specific sequences were amplified by PCR with 5 set of primers. Primers I to V designed for five chromosomes in Mic, respectively. The loci on chromosomes III, IV, and V appeared to be missing in Δ*MLH1*–B cells. I, 1.1 kb; II, 0.35 kb; III, 0.4 kb; IV, 1.2 kb; V, 0.9 kb. Arrow indicates band of Mic specific sequences.

To further investigate the function of Mlh1 in *Tetrahymena*, *MLH1* knock-out constructs were created (Fig 3A). Transformants were selected on the basis of blasticidin resistance and screened by PCR (Fig 3B). RT-PCR showed the mutants have no transcripts of *MLH1* (S2A Fig). *MLH1* gene was knocked out in Mac. *MLH1* knock-out strains proliferated normally during the vegetative growing stage. In *Tetrahymena*, the Mac is responsible for the somatic functions of the cell, while the defect of the Mic does no impair cell viability. The germline Mic undergoes programmed chromosome breakage and massive DNA elimination to generate the somatic Mac. Specific primers can be used to limit amplification to sequences present only at specific sites in the Mic. The specific PCR products amplified from micronuclear template are not affected by the high concentration of macronuclear DNA present in total genomic DNA, nor by variations in the number of nontarget chromosomes in multiply nullisomic Mics [30]. With this PCR assay, we analyzed the loss of Mic chromosomes in Δ*MLH1* strains (Fig 3C). To further understand the extent of chromosome loss, we also checked other loci of the right and left arms of I chromosome and III chromosome. The results indicated the chromosomes were disrupted randomly (S3 Fig). Taken together, these results showed that Mlh1 is required for micronuclear chromosome integrity and stability in *Tetrahymena*.

### Mlh1 is involved in conjugation development

**Fig 4:**
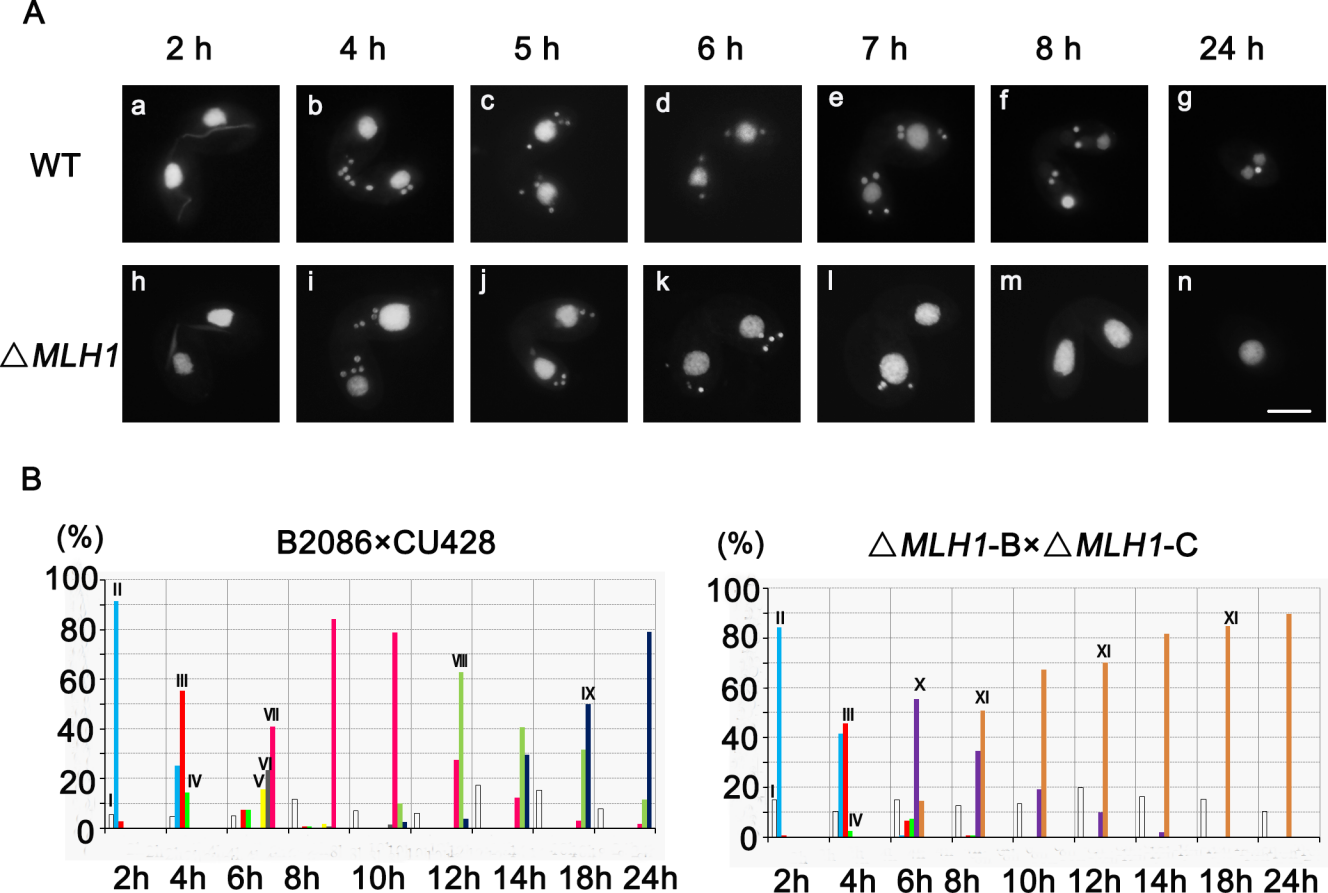
*MLH1* knock-out cells have defects in micronuclear development during the conjugation stage. (A) Mating cells were fixed and stained with DAPI. WT, wild type B2086 was mated with CU428. Δ*MLH1*, the mutant Δ*MLH1-*B was mated with Δ*MLH1-*C strains. a, h: crescent; b, i: meiosis II; c, j, k: pronuclear selection; d, postzygotic mitosis I; e, mitosis II; f, anlagen; g, 2Mac-1Mic; l, missing of “selected” pronucleus; m, pairs without Mics; n, single cell without Mics. Scale bar, 10μm. (B) Percentage of different developmental stages during the conjugation stage (n>200) in WT cells and Δ*MLH1* mutant cells, respectively. I, single cells; II, pair formation; III, crescent; IV, meiosis; V, pronuclear selection, post-meiotic mitosis, exchanged, and fuse; VI, postzygotic mitosis; VII, anlagen; VIII, pair separation with anlagen; IX, 2Mac-1Mic; X, pairs without Mics; XI, single cell without Mic.

**Fig 5:**
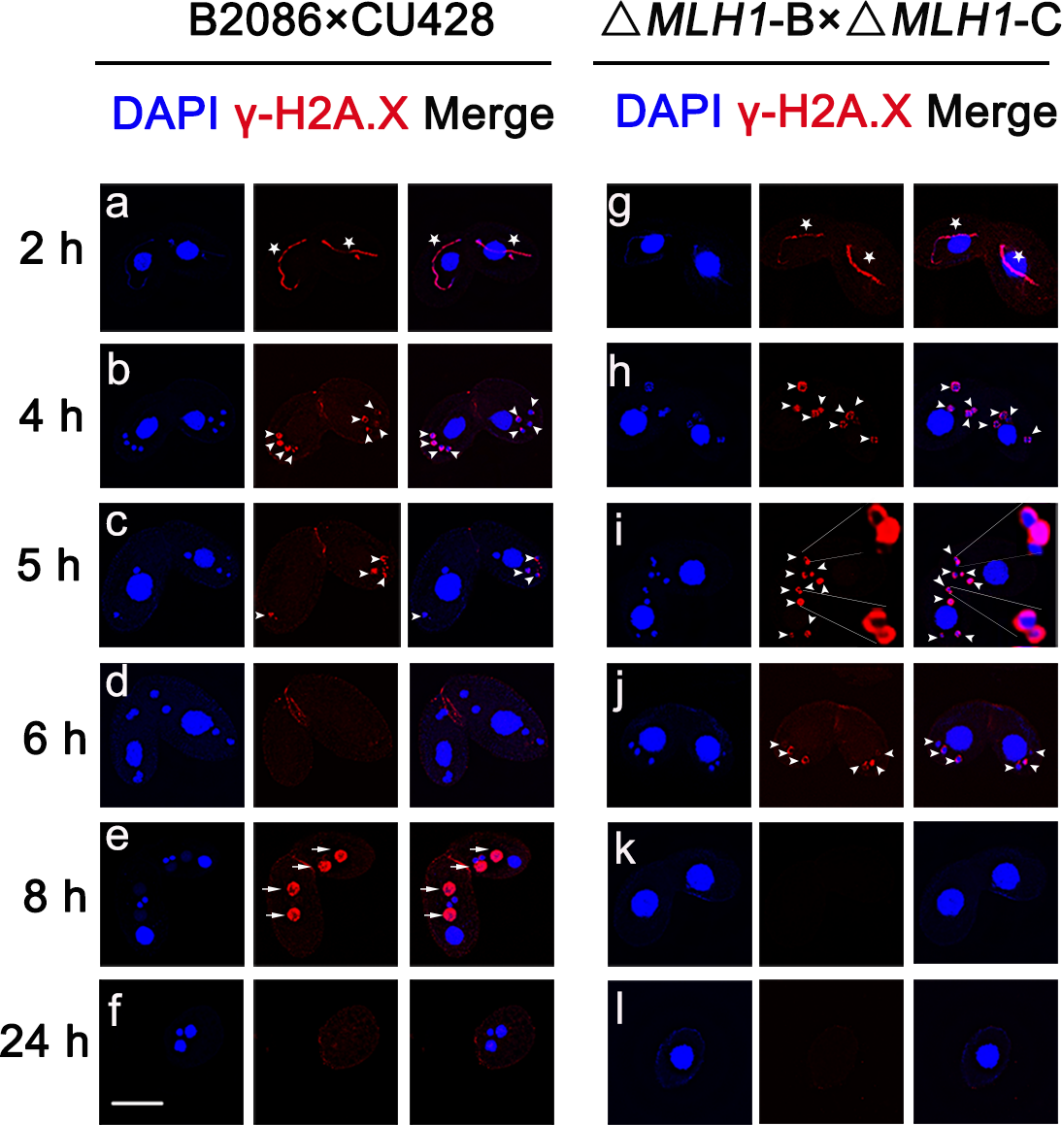
Mic DSBs failed to repair in *T*. *thermophila*. Immunofluorescence staining of γ-H2A.X in Mic. Anti-γH2A.X was used as an indicator of DNA double-strand breaks (DSBs), DAPI was used to stain nuclei. a, g: crescent; b, h: post-meiosis II; c: “selected” pronuclei; d: postzygotic mitosis; e: anlagen; f: exconjugant; i: unrepaired “selected” pronuclei; j: missing of “selected” pronucleus; k: pairs without Mics; l: single cell without Mics. Aterisk indicates crescent, arrowhead indicates Mic during meiosis, and arrow indicates anlagen. Scale bar, 10 μm.

Although knocking out *MLH1* led to micronuclear chromosome defect, Mlh1 is not essential for *Tetrahymena* vegetative growth [13]. Damaged micronuclei are known to impair sexual conjugation development. To investigate whether micronuclear damage in Δ*MLH1* cells would have similar effect, sexual development progress of Δ*MLH1* was analyzed. In WT, a remarkable feature of *Tetrahymena* meiosis is the extreme elongation of the mic during meiotic prophase. Δ*MLH1* mating cells failed to complete sexual development (Fig 4A) and the developmental progress stopped around zygotes formation stage (Fig 4B). The proteins and small RNAs transfer between two mating cells in *Tetrahymena*. The Δ*MLH1* cells were then mated with WT cells. Although WT cells failed to completely rescue the mutant cells, WT cells indeed improved normal phenotype in Δ*MLH1* cells (S2 Table). The result strongly argues that at least part of the meiotic phenotype in Δ*MLH1* cells are attributable to the lack of Mlh1 during meiosis, but not DNA defects accumulated during vegetative growth. In chicken DT40 cells, loss of histone H1R impaired sister chromatid exchange and accumulated IR-induced chromosomal aberrations at the G2 phase [31]. To further determine whether the abnormal conjugation development involved Mic DNA damage in Δ*MLH1* cells, DSB was evaluated by γ-H2A.X staining. In WT cells, γ-H2A.X staining was observed at the crescent-shaped Mics (Fig 5A), early four pronuclei (Fig 5B), and anlagen (Fig 5E). γ-H2A.X signal disappeared in successfully selected pronuclei (Fig 5C), while other three unselected nuclei maintained the signal until they were degraded (Fig 5C). However, four pronuclei were found to have γ-H2A.X signal at nuclear selection stage in Δ*MLH1* cells until they were all degraded (Fig 5H). The mating Δ*MLH1* cell failed to select functional pronuclei or the selected pronuclei failed to repair (Fig 5I). Four pronuclei disappeared at the same time (Fig 5J). The mating cells remained only their parental Mac and lost their Mics (Fig 5K and 5L).

### Overexpression of Mlh1 leads to abnormal conjugation

**Fig 6:**
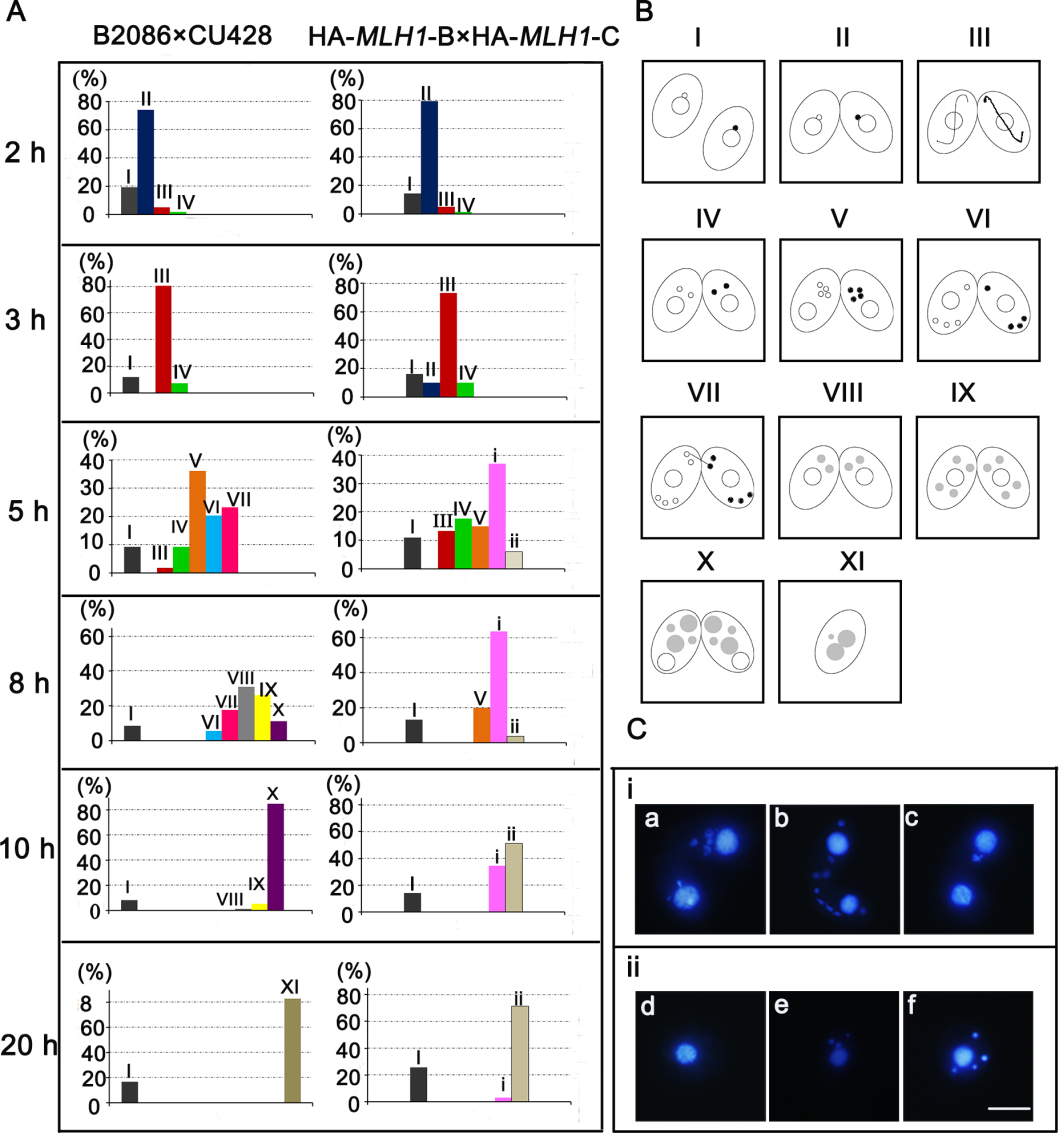
Overexpression of Mlh1 leads to defects during conjugation. (A) Percentage of different developmental stages during conjugation. I: single cell; II: pair formation; III: crescent; IV: meiosis I; V: meiosis II; VI: pronuclear selection; VII: post-meiotic mitosis; VIII: postzygotic mitosis I; IX: postzygotic mitosis II; X: anlagen; XI: 2Mac-1Mic. i: abnormal pairs; ii: abnormal signal cells (n>200). (B) Schematic representation of the developmental cells during conjugation. (C) A representative nuclear abnormal cell: (a) looser Mics; (b) irregular Mics; (c) loss of Mics; (d) separated cell with only one Mac; (e) one cell with three Mics; (f) one cell with four Mics. Scale bar, 10 μm.

Previously studies have showed that overexpressed HmgB3 abnormally binds with the chromatin structure of the developing MACs, which leads to the abortion of developed progenies [19]. To explore whether overexpressed Mlh1 also affect sexual development, Mutant and WT cells were treated with 0.15 μg/mL cadmium chloride, respectively. HA-*MLH1* was overexpressed under *MTT1* promoter with Cd^2+^ induction, its level at about 10 times that of WT cells (S2B Fig). Overexpression of *MLH1* abolished the formation and development of zygotic nuclei, and arrested conjugation progress (Fig 6A), as illustrated by the schematics for the *Tetrahymena* conjugation development stages (Fig 6B). Furthermore, overexpression of *MLH1* led to irregular Mics during the conjugation stage (Fig 6C).

### Functional compensation of Mlh1 and HmgB3

**Fig 7:**
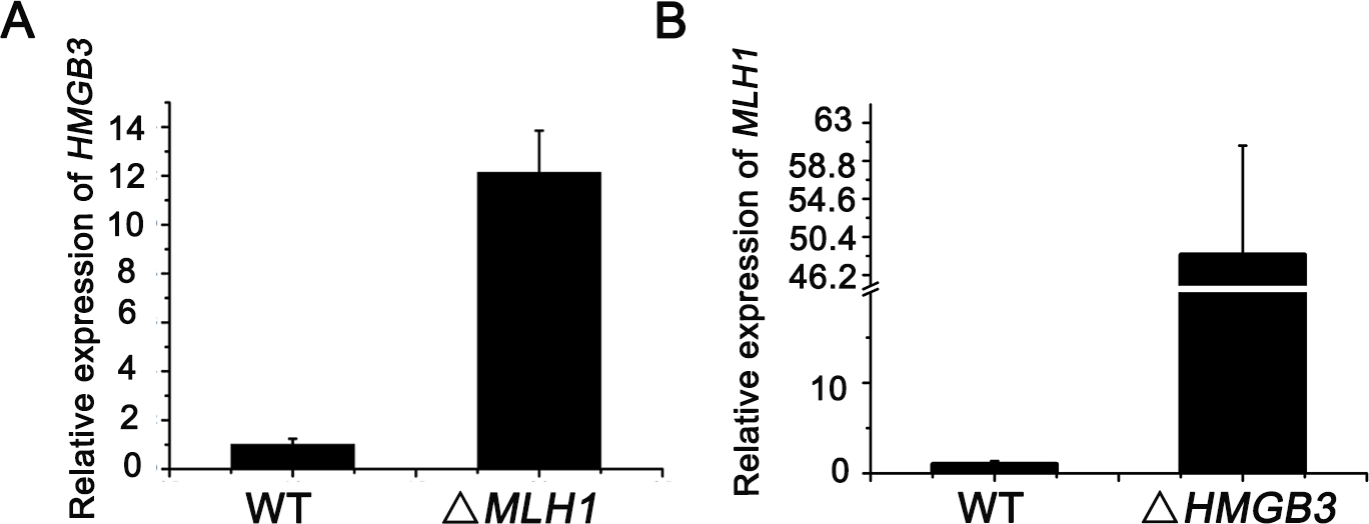
Functional compensation of *MLH1* and *HMGB3*. (A) RT-PCR analysis of *HMGB3* in the Δ*MLH1* strains. (B) RT-PCR analysis of *MLH1* in the Δ*HMGB3* strains. Total RNA was isolated from conjugating cells after mixing for 4.5 h. 17S rRNA was used as the internal control. Each reaction was performed in triplicate.

*MLH1* and *HMG-B3* have similar expression profiles. Furthermore, Mlh1 and HmgB3 have similar primary structure characterization. To explore whether there is functional compensation, the expression levels of *MLH1* and *HMGB3* were evaluated in different mutants. RT-PCR analysis showed that expression level of *HMGB3* increased in the Δ*MLH1* strains (Fig 7A). In our previous study, we found that knocking out *HMGB3* yielded no significant phenotype, but the expression level of *MLH1* was upregulated. Here, we also found the expression level of *MLH1* increased in the Δ*HMGB3* cells (Fig 7B). *HMGB3* knockout mutants had no obvious defects, but we found *MLH1* and *HMGB3* double knockout strains had more serious cytological defects than *MLH1* single knockout strains. These results suggest that HmgB3 and Mlh1 could have functional compensation in *T*. *thermophila*.

## Discussion

Histone H1s are multifunctional proteins found in various organisms [32]. *Tetrahymena* Mic contains five linker-associated proteins (Mlh1, α, β, γ, and δ) which originate from the same gene and the same transcript. The *MLH1* gene is transcribed and translated into a polypeptide Mlh1 that is processed into four micronuclear linker histones, namely α, β, γ, and δ. Mlh1 is a precursor to α and β, and α is further processed into γ and δ, a progress that is physiologically and developmentally regulated [16]. Previous studies showed that there are abundant α components during the vegetative growing stage. The amount of preexisting α in Mics decreases during early conjugation stage. The newly synthesized Mlh1 and α are deposited in the Mics. After the beginning of new macronuclear development, Mic contains unusually high amounts of α. During the later stages of nuclear differentiation (14–16 h), the amount of Mlh1 and α is reduced. In contrast, β, γ, and δ reappear in micronuclear chromatin only after the differentiation of new macronuclei and new micronuclei [33]. Microarray data showed that the expression level of *MLH1* is lower in the growing and starvation stages. However, expression level of *MLH1* increased significantly during the early conjugation stage (S4 Fig). HA-Mlh1 localized in the Mics during the growing and starvation stages. It also localized in the Mic during the early conjugation stage and disappeared during the late conjugation stage. Preexisting micronuclear-specific histones are “cleared” from micronuclear chromatin during the early periods of conjugation [33]. Immunostaining of the Mlh1 hydrolyzed product showed that HA-α, HA-β, HA-γ and HA-δ only localized in cytoplasm in the vegetative growing stage (Fig 1G). It appears that α, β, γ, and δ from cytoplasm failed to transport into Mic during the vegetative growing stage. These results indicate that full length Mlh1 was translated and transported into the Mic and then hydrolyzed into different fragments in Mic. Meanwhile, we didn’t found the localization of Mlh1-Flag (Flag inserted into C-terminal of Mlh1) in Mic (S5 Fig), which means Mlh1 was hydrolyzed quickly. The full-length Mlh sequence is required for it to localize at Mic, the expressed fragments only localized in cytoplasm. It is possible that Mlh1 contain discontinuous nuclear localization signal. Replication-independent histone H1.X prefers CpG islands and has distinct genome-wide distribution patterns in human breast cancer. It is involved in the expression of genes related to cell movement and transport [34]. By contrast, replication-dependent histone H1.2 is enriched at chromosomal domains, lamina associated domain, and chromosomes that are localized at the periphery of nuclei, which happens during chromatin compaction [35]. During conjugation stage, HA-Mlh1 co-localized with spindle structure around the crescent-shaped Mic. HA-Mlh1 not only deposited in the chromatins, but also associated with spindle microtubules, which could stretch the meiotic chromosomes. In fact, we failed to observe the localization of Mlh1 expressed by the new Mac since HA-Mlh1 was only expressed by the parental Mac. To explore localization of Mlh1 in the new Mic in future studies, a germline HA-Mlh1 mutant could be used.

In mouse H1 mutant cells, each H1 variant is at least partially redundant for embryogenesis and cellular viability and the expression of each variant in mutant cells was increased to compensate for reduction in total H1 protein [36]. A 50% reduction in the total amount of histone H1 triple knock-out mice triggered embryonic lethality [37]. *MLH1* knockout has no cytological influence on Mic mitosis and Mac amitosis during the vegetative growing stage. However, *MLH1* knockout led to abnormal nuclear morphology and aborted *Tetrahymena* sexual development. During conjugation stage, Mic is extremely elongated during meiotic prophase. Mlh1 could play a more important role in the process. γ-H2A.X staining remained at the selected pronuclei until they were degraded, implying that selected pronuclei cannot be repaired in the absence of *MLH1*. H1 homolog Hho1p is inhibitory to DNA repair in *S*. *cerevisae* [38]. H1R integrates into homologous recombinant-mediated repair pathways at the chromosome structure level. Loss of histone H1R has been reported to impair sister chromatid exchange and to accumulate IR-induced chromosomal aberrations at the G2 phase, with mutants exhibiting increased sensitivity to DNA damage in chicken cells [31]. Post-translational modifications of linker histones are important marks for recognition by factors involved in maintaining genome stability in human cells. Histone H1 is required for K63-linked ubiquitination and RNF168-dependent protein retention at DSB sites, facilitating chromatin remodeling to allow efficient DNA repair. Numerous post-translational modifications have been mapped on different H1 isoforms [6]. Future studies should explore whether Mlh1 is also modified by different marks and directly involved the micronuclear recombination repair during *Tetrahymena* Mic meiosis.

Experiments involving overexpression of histone H1 variants have revealed functional differences between different isoforms. Overexpression of Hho1p enhanced methyl-methane sulfonate (MMS) sensitivity in yeast [38]. Overexpression of H1(0) in the mouse 3T3 cell lines resulted in increase in nucleosomal repeat length and decline in cell cycle progression [39, 40]. Overexpression of H1c accentuated the normal functions of H1c by making larger quantities of this protein available for binding to regions not normally occupied by this variant. This would “open up” extensive regions of the chromatin, making it readily accessible for the binding of trans-activating factors [40]. In the study, we found that overexpression of *MLH1* led to sexual developmental arrest in *Tetrahymena*. Mating cells showed looser chromatin and irregular Mic structure. Thus, overexpression of Mlh1 also could “open up” extensive regions of the chromatin and affect cell cycle progression. Since Mlh1 is processed into α, β, γ and δ fragments, the specific function of the hydrolyzed products needs to be explored in the future.

In summary, Mlh1 is an evolutionary specific histone H1 and is important for germline micronuclear chromatin stability and sexual development. Full length Mlh1 is necessary for its micronuclear localization. The function of Mlh1 not only depends on its hydrolysis, but also compensated by HmgB3. Normal expression level of Mlh1 is necessary for micronuclear chromosome integrity in *Tetrahymena*.

## Supporting information

S1 Table Primers used in this study. (DOC)

S2 Table WT cells rescue the *MLH1* knock-out mutants. (DOC)

S1 Fig Western blotting of HA-Mlh1, HA-α, HA-β, HA-γ, and HA-δ. (DOC)

S2 Fig Expression of *MLH1* during log phase in the *MLH1* mutants. (TIF)

S3 Fig Amplification of Mic specific sequences. (TIF)

S4 Fig Expression profile of *MLH1*. (TIF)

S5 Fig Localization of HA-Mlh1 and Mlh1-Flag. (TIF)
