# Supplementary material for: Micronucleus-specific histone H1 is required for micronuclear chromosome integrity in Tetrahymena thermophila
Source: PLoS One. 2017 Nov 2;12(11):e0187475. doi: 10.1371/journal.pone.0187475 (PMC5667856; doi:10.1371/journal.pone.0187475)
Supplement: S1 Table — (DOC) [file pone.0187475.s001.doc]

**S1 Table. Primers used in this study.**

| **Primer name** | **Sequence** | **RECS** |
| --- | --- | --- |
| KO-*MLH1*-5’-FW  KO-*MLH1*-5’-RV  KO-*MLH1*-3’-FW  KO-*MLH1*-3’-RV  KO-*MLH1*-JD-FW  KO-*MLH1*-JD-RV  OE-*MLH1*-δ-FW  OE-*MLH1*-δ-RV  OE-*MLH1*-γ-FW  OE-*MLH1*-γ-RV  OE-*MLH1*-β-FW  OE-*MLH1*-β-RV  *MTT1*-FW  *MTT1*-RV  I-FW  I-RV  II- FW  II- RV  III- FW  III- RV  IV- FW  IV- RV  V- FW  V- RV  1L-2- FW  1L-2- RV  1L-6- FW  1L-6- RV  1R-2- FW  1R-2- RV  1R-4- FW  1R-4- RV  3L-3- FW  3L-3- RV  3L-4- FW  3L-4- RV  3R-1- FW  3R-1- RV  3R-2- FW  3R-2- RV  RT-*MLH1*-FW  RT-*MLH1*-RV  RT-*HMGB3*-FW  RT-*HMGB3*-RV | 5’- TGAGCTCCTTTAGTTAAACTTACTGATTAAATTTTC-3’  5’- TGCGGCCGCGCAAATCTTTCATAAGTATTGTCAAATC-3’  5’- TCTCGAGGTTAATTAATTACAAAATAGTTGGATGG-3’  5’- TGGTACCGGCAAATATTATATCATAAAGCTTATCC-3’  5’- CGCTTAATGCTGCAGCTTTTCCC-3’  5’- CATAATGGTGAATATTTGTTAAAATACC-3’  5’- GGATCCATGGAATTAAGAACTAAGACATTAAAT-3’  5’- GGCGCGCCTCCTTTAGAAACAGAAGATTTTCC-3’  5’- GGATCCAGAACAAAATCCACTTCTTCAAAGAG-3’  5’- GGCGCGCCAGCTTCTTTCATAGAGTTTCTTCTAC-3’  5’- GGATCCAGAACAAAGAAAGCCAACAATAAATCAG-3’  5’- GGCGCGCCTTATTTTTTATTTGCCTTCTTGCCATAAG-3’  5’- GCTACGTGATTCACGATTTATGCAATG-3’  5’- CGAAACTGATTTTATGCAATTATGAATTAC-3’  5’- ATCCAATATTAGGAACTGAAGC-3’  5’- TTTAATCATCTTAAAACTGAAGC-3’  5’- CTACATTAAAAATGATAAAA-3’  5’- CATGCATTTTTAATTTTGAG-3’  5’- AGATTAAACATAAGGATTCAAAC-3’  5’- TTGGTTATCTTTTAGTAAAGTTTG-3’  5’- AATAGAATAGCGACCATTAG-3’  5’- ATACTGATTTTTGCAACAAC-3’  5’- CAATAATTTCAAAAAAATGG-3’  5’- TAAAAAAGCAGGATTACAAT-3’  5’- GGGTTTTAACTTATTTTTAA-3’  5’- ATAGCAAATTGTTATATAGA-3’  5’- TTGATGAAACTTTAACGAGTAA-3’  5’- TGATTTTAGTTATCAGAATTCATTT-3’  5’- ATAAACCGCTTTTTAACTTTAG-3’  5’- TCTATTATGTTATTATGATGTAACG-3’  5’- AATCTCTTATATATTTTCAACCTTAG-3’  5’- ACCACAAAAGTAAGTTAAGTACG-3’  5’- TCAAAATTGGCTTTTAATTTC-3’  5’- AAAATAATTCAAACTCTTCTCAAC-3’  5’- TTCTGTTATATTATGAATGTGGA-3’  5’- TCAAGTTTTATCAAACAAAATG-3’  5’- GAATTTATATTATTTTAAGTTTCAACCC-3’  5’- AAATTATCTTTTTCTTTTGCTATC-3’  5’- AATAGCAGAATTTGTTCTTTGA-3’  5’- TGTTTTGACTTTAATTTAACACTG-3’  5’- GAGCTGATTCCTCAGCTTCTC-3’  5’- CTGCTTGAAGAGCGGGTACC-3’  5’- GAGTGGAATGAGTAGCATGCTCCTG-3’  5’-GCTGAAGCCTTCTTTTAAGAGGCCC-3’ | *Sac I*  *Not I*  *Xho I*  *Kpn I*  *BamHI*  *Asc I*  *BamHI*  *Asc I*  *BamHI*  *Asc I* |

Restriction endonuclease sites: *Bam*H I GGATCC; *Asc* I GGCGCGCC; *Not* I GCGGCCGC; *Sac* I GAGCTC; *Xho* I CTCGAG; *Kpn* I GGTAC. The sequence underlined indicates restriction endonucleases site.
