# Supplementary material for: Micronucleus-specific histone H1 is required for micronuclear chromosome integrity in Tetrahymena thermophila
Source: PLoS One. 2017 Nov 2;12(11):e0187475. doi: 10.1371/journal.pone.0187475 (PMC5667856; doi:10.1371/journal.pone.0187475)
Supplement: S2 Table — (DOC) [file pone.0187475.s002.doc]

**S2_Table. WT cells rescue the *MLH1* knock-out mutants**.

| **Type of mating cells** | **Pair formation %**  **(2 h)** | **Anlagen %**  **(8 h)** | **2Mac-1Mic %**  **(24 h)** |
| --- | --- | --- | --- |
| **WT-CU428×WT-B2086** | 91.57 | 84.17 | 79.02 |
| **Δ*MLH1*-B×Δ*MLH1*-C** | 84.20 | 0 | 0 |
| **Δ*MLH1*-C×WT- B2086** | 80.19 | 66.82 | 54.64 |

More than 200 pairs or exconjugants were counted for each instance.
