# Supplementary material for: Micronucleus-specific histone H1 is required for micronuclear chromosome integrity in Tetrahymena thermophila
Source: PLoS One. 2017 Nov 2;12(11):e0187475. doi: 10.1371/journal.pone.0187475 (PMC5667856; doi:10.1371/journal.pone.0187475)

**S1_Fig. West*ern* blotting of HA-Mlh1, HA-α, HA-β, HA-γ, and HA-δ** Total histones were extracted from log-phase of WT, HA-Mlh1, HA-α, HA-β, HA-γ, and HA-δ mutants, respectively. The extracted histones were separated by 12% SDS-PAGE. HA-Mlh1, HA-α, HA-β, HA-γ, and HA-δ were detected by anti–HA tag antibody. Arrows indicate HA-Mlh1, HA-α, HA-β, HA-γ, and HA-δ, respectively.


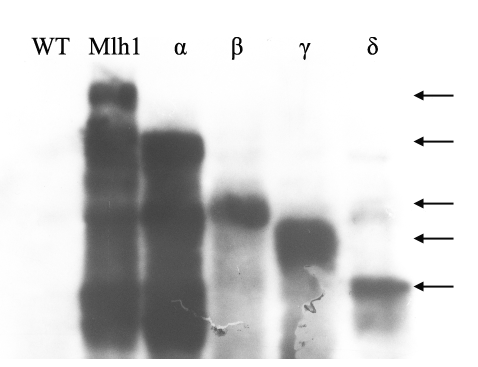

Supplement: S1 Fig — (DOC) [file pone.0187475.s003.doc]
