# Supplementary figures and images for: Micronucleus-specific histone H1 is required for micronuclear chromosome integrity in Tetrahymena thermophila
Source: PLoS One. 2017 Nov 2;12(11):e0187475. doi: 10.1371/journal.pone.0187475 (PMC5667856; doi:10.1371/journal.pone.0187475)

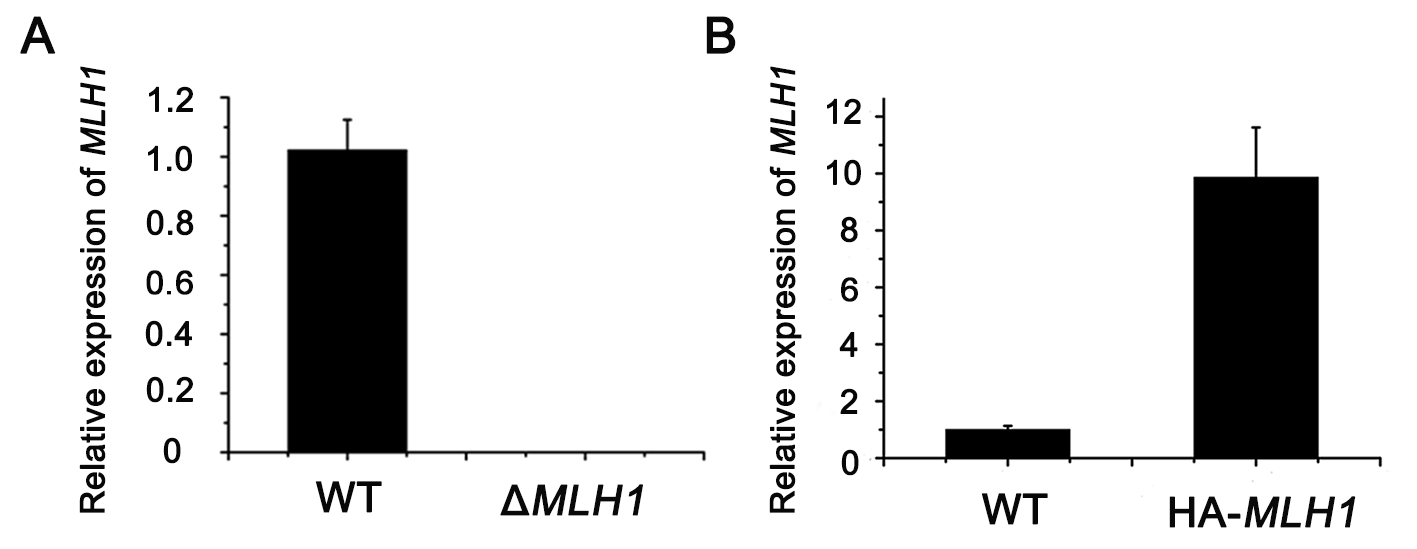

Supplement: S2 Fig — (TIF) [file pone.0187475.s004.tif]

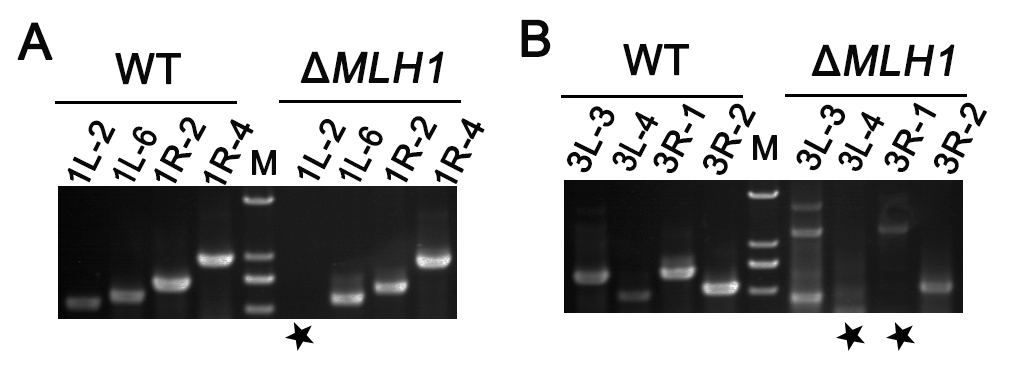

Supplement: S3 Fig — (TIF) [file pone.0187475.s005.tif]

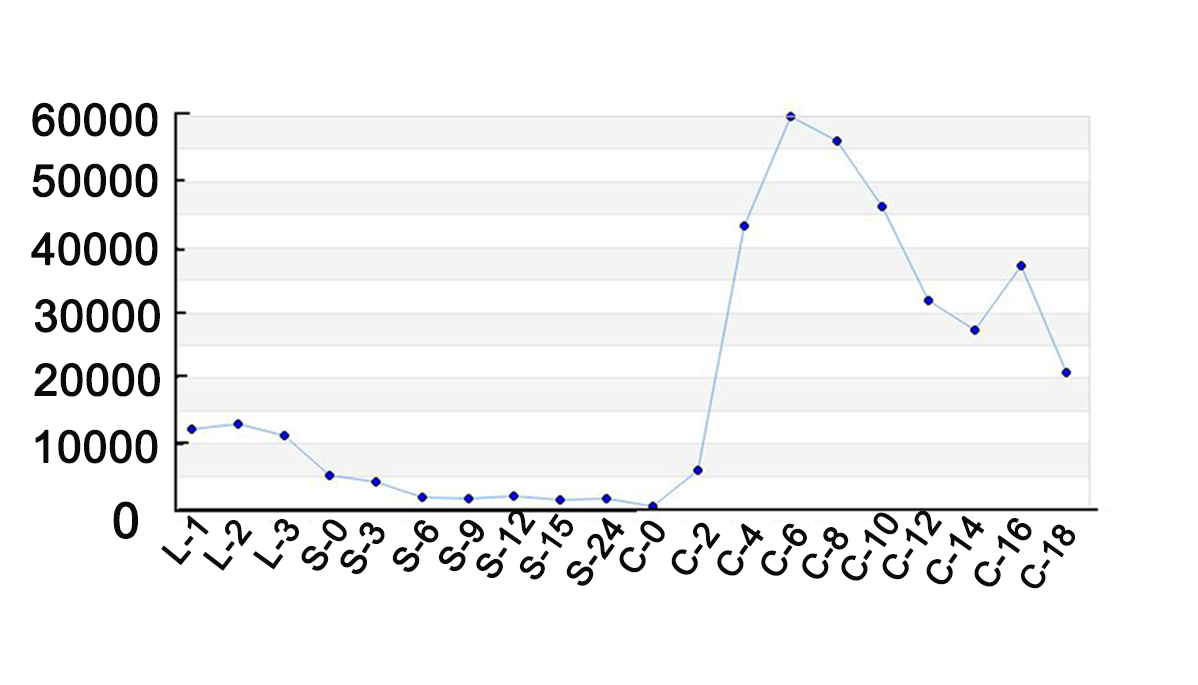

Supplement: S4 Fig — (TIF) [file pone.0187475.s006.tif]

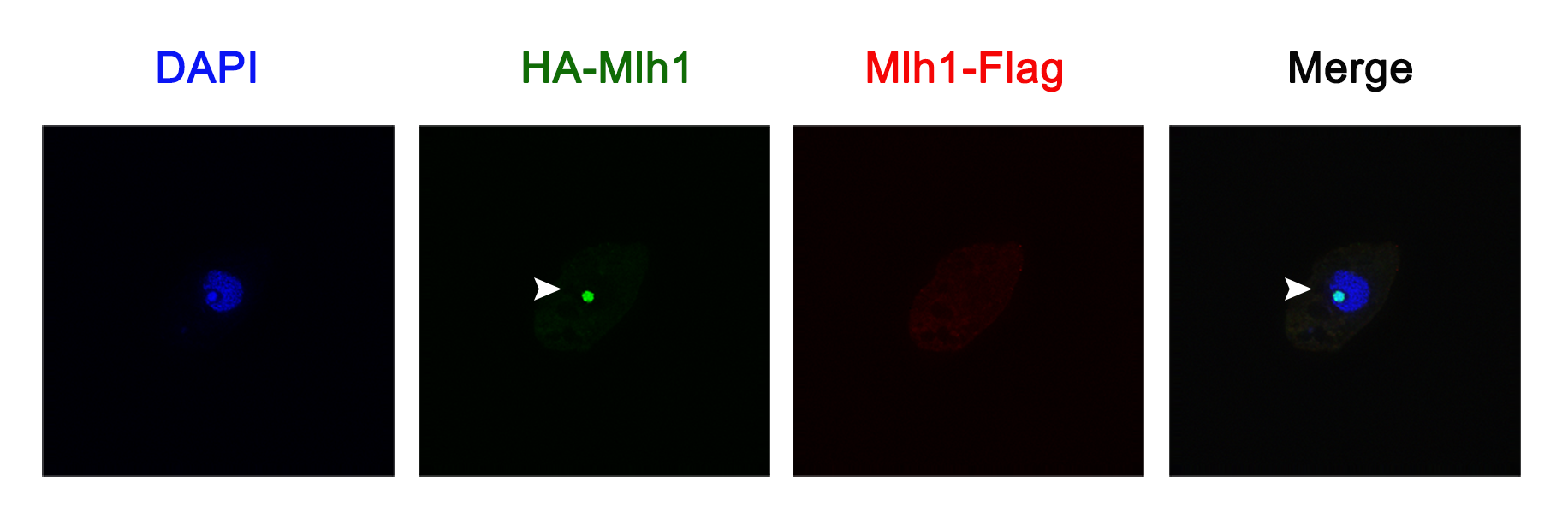

Supplement: S5 Fig — (TIF) [file pone.0187475.s007.tif]
